# Transactions Atlanta Obstetrical Society

**Published:** 1893-10

**Authors:** E. C. Davis

**Affiliations:** Secretary; Atlanta, Ga.


### *SOCIETY REPORTS.*

### TRANSACTIONS ATLANTA OBSTETRICAL SOCIETY.

Atlanta, Ga., August 10, 1893.

President W. A. Crowe in the chair.

A paper was read by Dr. V. O. Hardon upon the subject

### INDICATIONS FOR INDUCTION OF ARTIFICIAL ABORTION.

(SYNOPSIS.)

This is a subject with which every physician meets in his practice and there is no more important question to decide than that concerning the sacrifice of one or the other of two lives.

Abortion must be considered to mean the expulsion of the ovum occurring before the period of viability is reached by the child, although some authorities consider it to be the expulsion of products of conception before the end of the third month; a miscarriage after the fourth month to end of seventh; a premature labor from end of seventh to the term of pregnancy. The former classification is the one most convenient. To bring on labor after the seventh month is a very different procedure from its induction in the earlier months of gestation and places the two in very different categories.

The Divine fiat "Thou shalt not kill" is as pressing upon the physician as upon others. But the general consensus of opinion admits the justifiableness of abortion under certain circumstances. The life of an unborn infant is of less value than that of a mature woman. The Roman Catholic church, unless I have been incorrectly informed, forbids the taking of human life under any circumstances. Few physicians, however, even members of the Catholic church, hold to that view. The consensus of opinion already mentioned is unquestionably right.

*Indications which call for the Induction of Abortion.*—Abortion is justifiable only when the life of the mother is in jeopardy. This means a jeopardy which ceases at the termination of labor. Many men practice for a lifetime without meeting a case of this kind; they usually fall to the lot of the specialist.

The most common trouble which threatens the life of the woman is the vomiting of pregnancy. No condition calls for more impartial and discriminating judgment than these cases. Most all women vomit more or less, anyway, during pregnancy, and as one-half would prefer not to be pregnant at all, they argue so strenuously and artfully, magnifying their symptoms so much, that unless you are on your guard you may be deceived by them. I have had husbands come to me with the most exaggerated reports of vomiting of their wives, and when the wives were seen they failed to show signs of such extreme disturbance.

There is every gradation in the vomiting of pregnancy, from slight nausea to that which threatens the life of the woman. I have seen some women more nauseated and bear up better than others who did not suffer so much with nausea and vomiting. In other words, some women bear up under the nausea better than others. I do not know what this difference is dependent upon, but nervous susceptibility has something to do with it. The lower classes, negroes, etc., are not so subject to this trouble, while the higher classes, those who have been pampered and of more nervous texture, are more susceptible.

The symptoms which indicate pernicious vomiting are characteristic at first. The patient vomits for a few days and becomes so much exhausted as to have to take to her bed. The pulse rises, tongue becomes dry, cracked and coated, entering upon typhoid condition (the term typhoid is a very indefinite one) and delirium followed by death unless something is done. These women lose strength very rapidly and impressions on the nervous system and vitality are very noticeable.

The differences between the gradations are sharply drawn, so that I often have been able to predict at the outset whether the case will prove one which will require the induction of abortion. When the patient has reached the point of danger, she has gone too far; we should forestall this stage. When the woman is confined to bed with pulse of 120 or above, temperature 100° F. or more, if vomiting is not controlled by some measures, I consider it time to resort to operative measures. Even if the patient's strength be not much exhausted, but she be unable to retain anything upon the stomach, with pulse and temperature rising, then should we resort to operative procedures. I have seen one case die in which operative measures were put off too long.

*Indications for Induction* are: Inability to retain anything in the way of food, pulse rising above 120 and this condition remaining and resisting treatment.

The period to which pregnancy has advanced modifies the treatment very much. Some vomit the first month. If one of these patients develops the type of vomiting we have been considering, viz., pernicious vomiting, the conditions would be very different from those of a woman who had only two or three weeks longer to suffer from nausea. We all know about when vomiting usually ceases.

I have not spoken of the remedies for treatment of nausea of pregnancy. I must say I have derived but little benefit from medicines. I have gotten some amelioration of symptoms by the use of oxalate of cerium and bismuth by stomach, by local treatment through vagina long continued, the treatment of endometritis by application of compound tincture of iodine to cervix and use of glycerine tampons, the correction of displacements and loosening up of adhesions. One case which I now recall with marked retroversion was relieved by the correction of displacement by properly applied tampons.

Another condition causes us to think of producing abortions—pernicious anemia. I have never seen a case of this kind and have derived my knowledge concerning this trouble from text-books on obstetrics. Our conservative friend, Dr. Richardson, read a paper at the Georgia State Medical Association advocating the induction of abortion in cases of albuminuria. I do not hold to this view, for I believe eclampsia might result after the induction of abortion.

In some cases of placenta previa it becomes necessary to induce abortion. A woman with placenta previa is constantly in danger, bleeding is severe, and often she is where she cannot procure the aid of a physician. In such a case an abortion is indicated. In eclampsia, before the period of viability of child, I would induce abortion.

*Means of Inducing Abortion.*—There are three methods in common use. During the first and second months, I would choose the method of dilating and introducing blunt curette, clearing uterine cavity speedily. At three months, dilate and introduce curette forceps and remove contents. In both cases wash out uterus thoroughly after removal of contents. At four or five months, would not use curette, but introduce flexible bougie into uterus, leaving not more than one-half inch of bougie in vagina, then insert vaginal tampon of cotton. This is by far the best method after three and one-half months.

DISCUSSION.

DR. KIME: Dr. Hardon has pretty well covered the field and my ideas are in accord with his. In producing abortion we take into consideration the question of life. In placenta previa centralis the probability of delivering a viable child is not encouraging. This also is true in deformities of the pelvis and in the presence of tumors in certain sections. These are questions which confront the specialist oftener than the general practitioner. The methods described for inducing abortion up to the fourth month are such as I advocate. Later I should depend on injection of sterilized glycerine or introduction of bougie under antiseptic precautions.

DR. DUNCAN: I have had only two cases of placenta previa. In one I had no dilator, so used tampon and left patient to procure dilator. Was called back and found patient in labor. I had one other which was a central implantation of placenta. Mother did well; children both still born. My views in reference to nausea are in accord with those expressed by Dr. Hardon. I have gotten good results from use of carbolic acid applied to cervix, followed by glycerine tampon. One-fourth minim doses of fluid extract of ipecac sometimes yield very good results.

DR. CROWE: I have been very much pleased with the discussion to-night. The time and necessity for us to induce an abortion requires careful consideration and much discrimination. The common loose way of bringing on abortion under the slightest pretext is becoming a growing and serious evil of the day. As Dr. Hardon has stated, the majority of the women do not wish to become mothers, and as a result of this, we find that every city of any size has what are termed professional abortionists who coin money and have a large clientele, notwithstanding its direct violation to both the moral and State laws. This is a subject of universal interest to the profession at large, and the reports of this Society will be read with interest by all under whose notice it may come; therefore, we should be clear cut and explicit in regard to the indications for such a procedure, and I for one wish to join in the sentiment expressed here to-night and put myself on record as only being in favor of such a step where the safety of the mother is absolutely at stake. I was in hopes that Dr. Hardon would take up for consideration the size of neoplasm necessitating the induction of abortion. I had a case in which I detected a fibroid about the size of an orange during the earlier stages of the gestation. This case gave me considerable uneasiness in regard to its possibly complicating the birth of the child, but it never gave her any trouble whatever. She has since given birth to a second child, although the tumor is the size of a cocoanut. I also wanted to know his views in cases where we have strong evidence of malignant disease of the cervix. My views in regard to albuminuria are in full accord with those expressed in the paper to-night. I have never had a case to die from albuminuria of pregnancy. About three months ago I was called to see a patient who was between seven and eight months advanced in pregnancy. She was very edematous; eyesight so much affected that she could not bear a strong light or recognize any one a short distance off. The urine showed seventy-five per cent. *by volume* of albumin with heat and nitric acid. At the time of her confinement, about five weeks afterwards, the urine showed only twenty-five per cent. *by volume* of albumin. She had improved steadily under treatment and had an uneventful delivery, except perhaps some tendency to uterine inertia. The question of bringing on premature labor suggested itself in this case when I first saw her, but her condition was such that I feared the consequences, so I gave her the advantage of the doubt. It is wonderful the amount of tolerance in these cases, and I am inclined to believe that in most cases we can so improve the condition of the patient by proper treatment as to enable them to go to full term.

DR. HARDON: A word about tumors and contracted pelves. In cases of contracted pelves, I should elect Cesarean section under the present light of advancement in abdominal surgery. There are very few tumors that interfere with labor; a tumor must be very low down or very large to interfere materially with labor. Ovarian cysts can be tapped. I had one case in which I discovered fibroid tumor at fourth month, which was producing some disturbance. I opened abdomen and removed a pedunculated fibroid, and gestation went on uninterruptedly. Ovariotomies have been performed without interrupting gestation. A short time ago I was called to see a case with pain, hemorrhage and the passage of a membrane. The presence of an enlargement outside of uterus was detected. I diagnosticated this an ectopic pregnancy, and opened abdomen, finding the fetus in uterus, but the tubes matted and bound down by adhesions. I broke up the adhesions and the woman is now going along uninterruptedly. In cases of tumors at full term, I should prefer Cesarean section.

DR. KIME: In removing tubes and ovaries and tying them off close up to uterus, abortion is more likely to be produced than in abdominal sections for other conditions.

DR. HARDON: I don't see what would be gained by the induction of labor in malignant disease of cervix. This belongs in same category with contracted pelvis. As the mother's life cannot be spared more than a few months, I think the child should be given the best chance for its life.

DR. KIME: I had a case in which pregnancy produced retinal hemorrhages with almost complete loss of vision. The eye specialist, a physician of recognized ability, advised induction of abortion in case pregnancy should occur again. Would such a procedure be justifiable?

DR. CROWE: I was consulted about a patient who, during the period of pregnancy, is so addicted to the use of both whisky and opium as to make a perfect wreck of herself. It is also claimed that she is suffering from some mental derangement. She is the mother of three children. This condition has grown worse with every pregnancy. Her husband is extremely anxious that something should be done. What should be done under such circumstances?

DR. HARDON: I am acquainted with case mentioned by Dr. Crowe. The patient became addicted to opium habit by taking it for relief of dysentery before marriage. She was cured of the habit, but when she became pregnant she lost her power of will to resist, and began its use again. After delivery she was sent to a sanitarium and cured of the habit. In the second pregnancy the same state of affairs obtained; she took large quantities of morphine and whisky. All sharp instruments had to be secreted from her, for she showed maniacal and suicidal tendencies. This is a case in which, after due deliberation, I advised the induction of abortion. During the interim between pregnancies she is rational and very desirous to overcome the objectionable habits.

REPORT OF CASES.

DR. KIME: CASE 1.—Was called by Mrs. Dr. S. to see a primipara, colored, age sixteen years. Had been in labor three days, with but very few severe pains. Head well impacted in pelvis, producing such pressure that bladder could not be voluntarily emptied nor by use of catheter, as it could not be carried into bladder. Three different pairs of obstetric forceps were applied but would not hold position on head, slipping off when any traction was applied. The child being dead, we decided to perforate, which resulted in the escape of between two and three pints of fluid, after which the child was delivered without difficulty. It was hydrocephalic and was not detected at first on account of impaction in pelvis.

CASE 2.—Called by Dr. P. to see a multipara in labor suffering with hemorrhage, having had two or three previous hemorrhages. The cervix being rigid and only dilated to about the size of a silver quarter, I decided to tampon. After tamponing antiseptically, I left with instructions that I would return in about four hours. On returning found patient had vomited and in straining had forced out tampon and was having free hemorrhage. I lost no time in disinfecting my hand and carrying it up into uterus, performed podalic version, bringing extremities down into cervix which acted as a tampon, then completed delivery with less haste.

On delivering placenta found a battledoor attachment of cord, which attachment was immediately over the os uteri when placenta was *in situ*. Hand passed through margin of placenta just outside of insertion of the cord. Saline solution was thrown into rectum, stimulants, tonics and liquid nourishment ordered. When delivered, pulse was 180 and scarcely perceptible, temperature subnormal. Patient had a slight elevation of temperature and was dismissed by the physician in charge on the tenth day. Was sent for on the fifteenth day after labor, stating that patient was "suffering pain and swelling of left foot and leg." On arrival we found patient suffering as stated, with temperature $104\frac{2}{5}°$ F., pulse 140, with inflammatory exudate in left broad ligament, tender on pressure, uterus large, but no special indications of peritonitis. Dilated cervix and washed out two or three ounces of retained secretions and *débris*. After each irrigation and disinfection of uterus, pulse would drop ten to fifteen beats and temperature one degree or more. This procedure, with hot water vaginal douches and the usual constitutional treatment for such cases, was carried out for four days with decided improvement in pelvic condition and some amelioration of constitutional symptoms, but having to prepare for an abdominal section on another case had to retire from this case, after which she refused to carry out any further treatment, local or constitutional. The last report from case, three months after, was that she was yet an invalid. At the time, patient was not able to stand the shock of an operation, neither did I consider one justifiable just at that period.

DR. CROWE: In connection with Dr. Kime's cases, I wish to report a case of placenta previa that I was called to see in consultation. It was the variety known as placenta previa partialis. She was about six months pregnant. The os was dilated to about the size of a twenty-five cent piece when I saw her. She had had a considerable flow two weeks previous, without any pains whatever. She was put to bed and kept perfectly quiet. This second flow came on without any exertion. She had lost so much blood that we determined to turn and deliver as quick as possible. While I was dilating the os I was compelled to remove my hand on account of cramp. My patient came very near bleeding to death while my hand was out. While dilating I had controlled the bleeding completely by direct pressure on the portion of protruding placenta. Used saline solution in the bowel with gratifying results. The patient has made a good recovery.

E. C. DAVIS, Secretary.

---

### SUPRAPUBIC LITHOTOMY.

Lawson Tait thus describes the *technique* of this operation: "I make use of no precautionary measures or preparatory steps. I neither pack the rectum nor distend the bladder. I stand on the left of my patient and cut upward two inches and a half, starting immediately over the ridge of the pubic arch, exposing the tendon at one sweep. I then cut the tendon traversely over about one inch close to the bone, and cut it centrally for an inch and a half. I then pass my left forefinger between the bladder and the pubic arch and follow it with a pair of forceps. I gently rend the tissue till I can feel bladder wall. This can easily be determined by its peculiar feeling, and by the fact that once the forceps grip it they hold, and they do not hold merely cellular tissues. Having fixed one pair, I then fix another close to them. My assistant takes them and gently pulls them apart, as in abdominal section; a notch of the knife follows, and a rush of water declares the road into the bladder for the forefinger to be open. The rest is all finger work, and consists as in abdominal sections of a gentle, but firm extension of the opening into the bladder, till the lithotomy forceps can follow it. All my cases have recovered without complication, and though up to the present I have used a glass drainage tube, I am of the opinion that this will prove an unnecessary precaution, and that it will be safe to close the bladder by deep sutures."—*Med. and Surg. Rep.*

